# Mycorrhizal Effects on Growth and Expressions of Stress-Responsive Genes (*aquaporins* and *SOSs*) of Tomato under Salt Stress

**DOI:** 10.3390/jof8121305

**Published:** 2022-12-16

**Authors:** Sheng-Min Liang, Qiu-Shuang Li, Ming-Yang Liu, Abeer Hashem, Al-Bandari Fahad Al-Arjani, Mekhled M. Alenazi, Elsayed Fathi Abd_Allah, Pandiyan Muthuramalingam, Qiang-Sheng Wu

**Affiliations:** 1 College of Horticulture and Gardening, Yangtze University, Jingzhou 434025, China; 2 Botany and Microbiology Department, College of Science, King Saud University, P.O. Box 2460, Riyadh 11451, Saudi Arabia; 3 Plant Production Department, College of Food and Agricultural Sciences, King Saud University, P.O. Box 2460, Riyadh 11451, Saudi Arabia; 4 Division of Horticultural Science, Gyeongsang National University, Jinju 52725, Republic of Korea

**Keywords:** arbuscular mycorrhizal fungi, nitrogen balance index, PIPs, salt stress, SOS, TIPs

## Abstract

Environmentally friendly arbuscular mycorrhizal fungi (AMF) in the soil can alleviate host damage from abiotic stresses, but the underlying mechanisms are unclear. The objective of this study was to analyze the effects of an arbuscular mycorrhizal fungus, *Paraglomus occultum*, on plant growth, nitrogen balance index, and expressions of *salt overly sensitive* genes (*SOSs*), *plasma membrane intrinsic protein* genes (*PIPs*), and *tonoplast intrinsic protein* genes (*TIPs*) in leaves of tomato (*Solanum lycopersicum* L. var. Huapiqiu) seedlings grown in 0 and 150 mM NaCl stress. NaCl stress severely inhibited plant growth, but *P*. *occultum* inoculation significantly improved plant growth. NaCl stress also suppressed the chlorophyll index, accompanied by an increase in the flavonoid index, whereas inoculation with AMF significantly promoted the chlorophyll index as well as reduced the flavonoid index under NaCl conditions, thus leading to an increase in the nitrogen balance index in inoculated plants. NaCl stress regulated the expression of *SlPIP1* and *SlPIP2* genes in leaves, and five *SlPIPs* genes were up-regulated after *P*. *occultum* colonization under NaCl stress, along with the down-regulation of only *SlPIP1;2*. Both NaCl stress and *P*. *occultum* inoculation induced diverse expression patterns in *SlTIPs*, coupled with a greater number of up-regulated *TIPs* in inoculated versus uninoculated plants under NaCl stress. NaCl stress up-regulated *SlSOS2* expressions of mycorrhizal and non-mycorrhizal plants, while *P*. *occultum* significantly up-regulated *SlSOS1* expressions by 1.13- and 0.45-fold under non-NaCl and NaCl conditions, respectively. It was concluded that *P*. *occultum* inoculation enhanced the salt tolerance of the tomato, associated with the nutrient status and stress-responsive gene (*aquaporins* and *SOS1*) expressions.

## 1. Introduction

The greenhouse cultivation of horticultural crops has become a major mode, but irrational irrigation, the excessive application of fertilizers, and poor air mobility tend to cause salt accumulation and soil secondary salinization, leading to a decrease in the yield and quality of horticultural crops [1]. Therefore, soil secondary salinization has become a major factor in the occurrence of successive cropping obstacles in greenhouse horticultural crops [2]. The tomato is a worldwide important vegetable crop with a global annual production of nearly 1.78 × 10^8^ tons, and China ranks first in the world in terms of tomato cultivation areas and production [3]. Of the 1.1 million hectares of tomatoes grown in China, greenhouse tomatoes account for 58% [3]. In addition, cultivated tomatoes are moderately sensitive to soil salt levels and irrigation water [4]. As a result, it is key to find suitable methods to improve the salt tolerance of tomatoes under greenhouse conditions.

A class of beneficial fungi, arbuscular mycorrhizal fungi (AMF), exists around tomato roots [5], where they colonize roots and establish a mutualistic symbiosis that provides nutrients and water to the host tomato, thus promoting plant growth, yield, and fruit quality [6,7,8]. The presence of arbuscular mycorrhizae in tomato roots has been known to enhance the resistance of the tomato to biotic and abiotic stresses, including salt stress [9,10,11]. In the tomato, AMF mainly induces ion balance, photo-chemistry, and an antioxidant defense system in response to salt stress [12,13], while the underlying mechanism is not yet understood. Under salt stress, mycorrhizal fungal colonization dramatically elevates biomass production, reduces shoot Na^+^ concentrations, and does not alter shoot K^+^ concentrations of the tomato [9]. The change in Na^+^ and K^+^ concentrations is associated with the Na^+^/H^+^ antiporter *salt overly sensitive 1* (*SOS1*) gene, coupled with the synergistic effect of *SOS2* and *SOS3* [14]. There is no information on whether and how *SOS* genes of tomatoes respond to AMF colonization under salt stress.

Salt-stressed tomatoes represent higher leaf water potential and root hydraulic conductance under mycorrhizated versus non-mycorrhizated conditions [15]. Changes in water potential under salt stress are associated with expressions of *aquaporins* (*AQPs*), especially *plasma membrane intrinsic proteins* genes (*PIPs*) and *tonoplast intrinsic proteins* genes (*TIPs*) [16,17]. He et al. [15] also reported the down-regulation of a *TIP* gene and two *PIP* genes in a tomato under salt stress, accompanied by the up-regulation of only *SlAQP2*. In fact, in the Genome-Wide of tomatoes, Reuscher et al. [18] identified 47 *AQPs*, including 14 homologs in the *PIP* family and 11 homologs in the *TIP* family. The study of a single or small amount of *AQP* gene expression could not reveal the regulation effect of mycorrhizal fungi on host *AQPs*.

The objective of this study was to evaluate the effects of AMF on the growth, physiological activities, and the expression of stress-responsive genes (*SOSs*, *TIPs*, and *PIPs*) in tomato seedlings under salt and non-salt stress, which was expected to reveal the molecular mechanism of the salt tolerance of mycorrhizal tomato plants.

## 2. Materials and Methods

### 2.1. Preparation of Mycorrhizal Inoculums

An arbuscular mycorrhizal fungus used in this study was *Paraglomus occultum*, which was isolated from peach trees grown in saline soils (pH 8.15, organic carbon 8.5 g/kg, Olsen-P 9.81 mg/kg, and total P 59 mg/kg) in Beijing, identified at the morphological level, and kept in our laboratory [19]. The chlamydospores of *P. occultum* were solitary in the soil and (65−)75−100(−140) μm in diameter, or a few existed on the tip of the mycelium; the spore shape was globose or subglobose, with no auxiliary cells; the spore wall had two layers, 2.5 μm, all colorless and transparent; in Melzer’s reagent, the spores were orange [19]. In addition to morphological recognition, this fungus was also identified at the molecular level by Wang et al. [20] using the cad 5.1 primer (5′-GAAGTCTGTCGCAGTCTG-3′). The fungus had been preserved in the Bank of Glomeromycota in China (BGC), and its number was BGC BJ04B. The fungus was propagated in potted white clover (*Trifolium repens* L. var. Olwen) for approximately 10 weeks, and the root segments and potting substrates were collected as mycorrhizal inoculums, in which spore density was 15 spores per g inoculum.

### 2.2. Plant Culture and Salt and AMF Treatments

Tomato (*S*. *lycopersicum* L. var. Huapiqiu) seeds from Hezhiyuan Seed Industry Co., Ltd. (Weifang, China) were surface-sterilized in 75% ethanol and sown into 32-hole trays containing an autoclaved mixture of perlite, vermiculite, and peat with an environment of 28 °C/20 °C (day/night temperature, 16 h/8 h) and 80% relative humidity. After 20 days, the tomato seedlings were transplanted into pots containing 1.9 kg of an autoclaved mixture of soil and sand (3:1, *v*/*v*). After autoclaving (121 °C, 0.11 MPa, 2 h), the mixture properties were as follows: pH 6.0, 27.5 cmol/kg of cation exchange capacity, 41.9 mg/kg of NH_4_^+^-N, 74.3 mg/kg of NO_3_^−^-N, 0.50 g/kg of available K, and 46.2 mg/kg of Olsen-P. The sand was less than 0.5 mm in diameter, with 68% of SiO_2_. Meanwhile, mycorrhizal fungus-inoculated plants received an additional 120 g of *P*. *occultum* inoculums containing approximately 1800 spores, while uninoculated plants received the same amount of autoclaved *P*. *occultum* inoculums plus 2 mL of filtrate (25 μm) of the same weight of inoculums that had not been autoclaved, in order to re-establish the background microflora. The filtrate was applied into the rhizosphere of potted seedlings. Therefore, there was a total of 2.02 kg of materials in each pot, evenly distributed. These inoculated plants were then placed in a greenhouse for growth with a photosynthetic photon density of 900 μmol/m^2^/s, a day/night temperature of 28 °C/20 °C (16 h/8 h), and a relative air humidity of 70%. Potted soil water was controlled daily at 75% of the maximum soil water holding capacity. After one month, inoculated and uninoculated plants were subjected to 0 and 150 mM NaCl treatments. The concentration of 150 mM NaCl was achieved with gradual increases in a gradient of 50 mM NaCl per day to avoid salt shock. For the salt treatment, 100 mL of the set salt concentration was watered per pot at an interval of 3 days, maintaining a total of 10 irrigations. Then, all treated plants were harvested. At the end of the experiment, the pH value of the substrate was 8.3. As a result, the experiment included the following four treatments: the tomato plants not inoculated with *P*. *occultum* were subjected to 0 mM NaCl (Po^−^Na^−^); the plants inoculated with *P*. *occultum* were subjected to 0 mM NaCl (Po^+^Na^−^); the plants not inoculated with *P*. *occultum* were subjected to 150 mM NaCl (Po^−^Na^+^); the plants inoculated with *P*. *occultum* were subjected to 150 mM NaCl (Po^+^Na^+^). Each treatment was replicated six times and arranged randomly.

### 2.3. Variable Determinations

The potted mixture’s pH was determined using the pH meter (Sartorius Scientific Instruments Co., Ltd., Beijing, China). The cation exchange capacity of the substrate was determined by the NH_4_Cl-NH_4_OAc method [21]. The NH_4_^+^-N, NO_3_^−^-N, available K, and Olsen-P levels of the substrate were measured with a Soil Fertilizer Nutrient Tester (HM-TYD; Shandong Hengmei Electronic Technology Co., Ltd., Weifang, China) according to the user’s guidelines.

On the day of the harvest, plant height and stem diameter were measured directly, and then the chlorophyll index (Chi), flavonoids index (Flav), and nitrogen balance index (Nbi) (the ratio of Chi to Flav) were measured by a portable plant polyphenol-chlorophyll apparatus (Dualex Scientific+, Force-A, Orsay, France).

At harvest, the biomass of the whole plant was weighed. The roots were scanned with the Flatbed Scanner (V700, EPSON), along with the analysis of root length, surface area, and volume by the WinRHIZO pro software (2007c) (Regent Instruments Inc., Quebec city, Canada).

The 1–2 cm long root segments were collected and stained with trypan blue in lactic acid phenol solution for mycorrhizal staining [22], and then a further microscopic observation was performed. The colonization rate of mycorrhizal fungi in roots was estimated as the percentage of the root segment length colonized by mycorrhizal fungi versus the observed root segment length.

Then, the total RNA of leaf samples was extracted with the EASY spin Plus Plant RNA Rapid Extraction Kit (Aidlab, RN38, Beijing, China) according to the manufacturer’s protocol. After testing the integrity and purity of the obtained RNA, the RNA was reversely transcribed into cDNA using the PrimeScript^TM^ RT Reagent Kit with gDNA Eraser (TaKaRa, RR047A, Beijing, China). *SlPIP* and *SlTIP* gene sequences were obtained from the Solanaceae Genomics Network (https://solgenomics.net/, accessed on 15 July 2022), and *SlSOS1* and *SlSOS2* gene sequences were obtained from NCBI (http://github.ncbi, accessed on 15 July 2022). The online tool PrimerQuest^TM^ (http://sg.idtdna.com/primerquest/Home/Index, accessed on 15 July 2022) was used to design corresponding specific primers (Supplementary Material Table S1). A quantitative real-time PCR analysis was performed using the ChamQ Universal SYBR qPCR Master Mix (Vazyme, Q711) with β-actin as the housekeeping gene. The 2^−ΔΔCt^ method [23] was used to analyze the relative expression of genes, and the Po^−^Na^−^ treatment was used for the normalization.

### 2.4. Data Analysis

Sigmaplot 10.0 software was used for plotting. The experimental data were subjected to a one-way ANOVA using SAS 8.0 software, and then further tested for the significance of differences between treatments (*p* < 0.05) using the Duncan’s multiple range test.

## 3. Results and Discussion

### 3.1. Effects of NaCl on Root Colonization of Tomato by P. occultum

**Figure 1:**
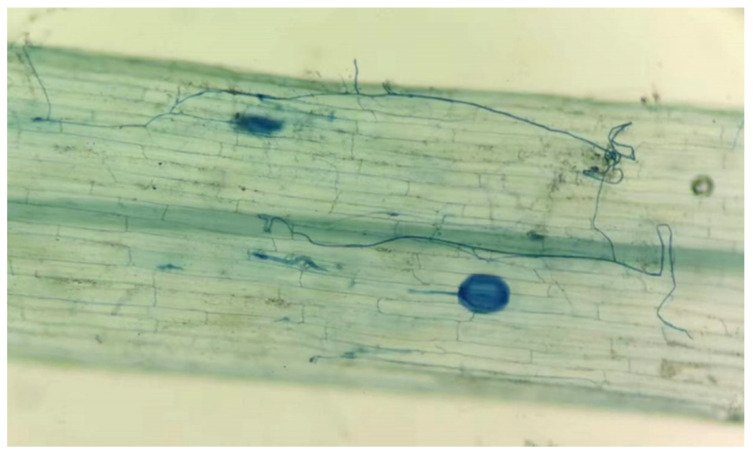
Root colonization of tomato plants by *Paraglomus occultum*.

No signs of mycorrhizal colonization were observed in tomato roots without *P. occultum* inoculation, while mycorrhizal fungal colonization was clearly visible in the roots of inoculated plants (Figure 1), with the root colonization rate ranging from 38.6% to 49.1% (Table 1). The treatment with 150 mM NaCl significantly inhibited the mycorrhizal fungal colonization in roots by 0.21-fold, compared to the 0 mM NaCl treatment. Similar results were observed in trifoliate orange seedlings colonized by *Funneliformis mosseae* exposed to 100 mM NaCl for 32 days [16]. The decrease in the root mycorrhizal colonization rate in response to NaCl stress may be attributed to the inhibition of spore germination and mycelial growth and the decreased transfer of plant carbohydrates to root mycorrhizae [24].

### 3.2. Effects of P. occultum on Plant Growth of Tomato Exposed to NaCl Stress

In the present study, NaCl stress somewhat inhibited plant growth performance as well as biomass production, especially in plant height and total plant biomass (Table 1). On the other hand, compared with the non-inoculation treatment, *P*. *occultum* inoculation also significantly increased the stem diameter and total plant biomass under non-NaCl stress conditions by 20.00% and 48.04%, respectively; *P*. *occultum* colonization also significantly increased plant height, stem diameter, and total plant biomass under NaCl stress by 40.51%, 36.84%, and 43.33%, respectively. Similarly, Ait-El-Mokhtar et al. [25] also reported positive effects of a mixture of AMF on the growth parameters of date palm seedlings under 240 mM NaCl stress conditions. The improvement of plant growth by mycorrhization under salt stress implied that mycorrhizal plants have greater adaptability of salinity than non-mycorrhizal plants, which is partly associated with the mycorrhizal enhancement of plant mineral element acquisition, maintenance of improved ionic balance, and tolerance indicators such as proline [25,26].

### 3.3. Effects of P. occultum on Index of N Balance, Chlorophyll, and Flavonoids of Tomato Exposed to NaCl Stress

**Figure 2:**
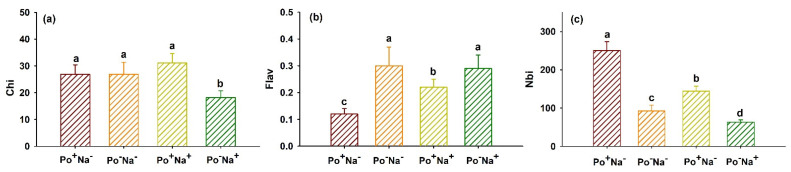
Changes in leaf chlorophyll index (Chi) (**a**), flavonoid index (Flav) (**b**), and nitrogen balance index (Nbi) (**c**) of tomato plants colonized by *Paraglomus occultum* under 0 and 150 mM NaCl stress. Data (means ± SD, *n* = 6) followed by different letters on the bar indicate significant differences among treatments at the 0.05 level. See Table 1 for abbreviations.

It is well-known that when plants are in a healthy state, their chlorophyll content is high, and relatively few polyphenols (flavonoids) are produced at this time; once plants are subjected to nitrogen deficit, chlorophyll synthesis is inhibited, and a large number of polyphenols (flavonoids) are produced, causing an imbalance of nutrients in plants [27]. Therefore, plant Nbi can quickly and effectively evaluate the nutritional status of plants. In the present study, NaCl stress significantly inhibited leaf Nbi in inoculated and uninoculated tomato plants as well as Chi in uninoculated tomato plants, but distinctly increased Flav in inoculated tomato plants, compared with non-NaCl stress (Figure 2a–c). On the other hand, *P*. *occultum* inoculation under non-NaCl stress significantly inhibited Flav by 60.00% but dramatically increased Nbi by 170.41%, along with no change in Chi, as compared with non-inoculation. Under NaCl stress, *P*. *occultum* inoculation significantly increased leaf Chi and Nbi by 70.88% and 127.80%, respectively, but also inhibited it by 24.14%, as compared with non-inoculation. Dai et al. [28] also reported that in tea, *Claroideoglomus etunicatum* inoculation significantly increased leaf Nbi and Chi under soil drought stress, as compared with non-inoculation. 

Based on our study, it can be understood that mycorrhizal plants promoted Nbi by increasing leaf Chi and decreasing Flav under NaCl stress, thus indicating that the nutritional status of mycorrhizal plants was significantly better than that of non-mycorrhizal plants under salinity. Earlier studies also observed greater nutrient levels in mycorrhizal versus non-mycorrhizal plants under salt stress, such as pistachio [29], wheat [30], and trifoliate orange [31].

### 3.4. Effects of AMF on PIPs’ Expressions in Leaves of Tomato under NaCl Stress

**Figure 3:**
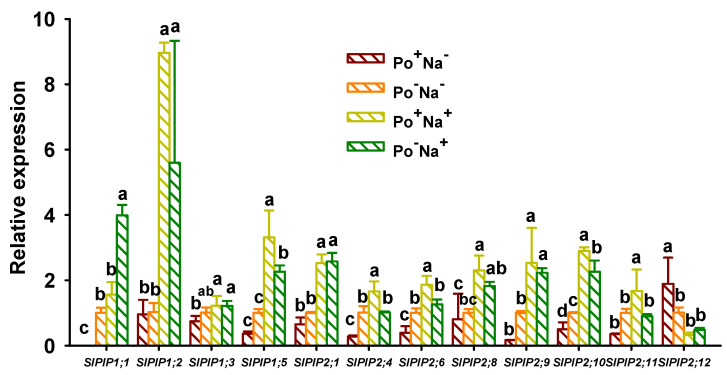
Changes in leaf *SlPIP1* and *SlPIP2* expressions of tomato plants colonized by *Paraglomus occultum* under 0 and 150 mM NaCl stress. Data (means ± SD, *n* = 3) followed by different letters on the bar indicate significant differences among treatments at the 0.05 level. See Table 1 for abbreviations.

Plant *PIPs* are divided into two classes, *PIP1* and *PIP2*, localized on the plasma membrane, where *PIP1* acts as water transport as well as root conductivity maintenance and stomatal conductance, but *PIP2* has a higher water transport activity than *PIP1* [32]. In this study, we found that NaCl stress up-regulated the expression of *SlPIP1* and *SlPIP2* genes in leaves of both inoculated and uninoculated plants in almost all cases, except for no change in the expression of *SlPIP1;3*, *SlPIP2;4*, *SlPIP2;8*, *SlPIP2;11*, and *SlPIP2;12* in uninoculated plants (Figure 3). This reveals that *PIPs* of AMF-inoculated tomato were able to rapidly up-regulate their expression and thus promoted water uptake and root hydraulic conductance; therefore, inoculated plants have a higher salt tolerance than uninoculated plants. On the other hand, *P*. *occultum* colonization under non-NaCl stress conditions barely altered the expression of *SlPIP1* and *SlPIP2*, except for the down-regulation in *SlPIP1;1*, *SlPIP2;4*, and *SlPIP2;10*, and the up-regulation in *SlPIP2;12*. This is because under non-salt stress, there is sufficient water in the soil surrounding roots, and plants can obtain enough water by relying on roots, thus reducing the regulatory role of mycorrhiza in *AQPs* [24,33]. In contrast, under NaCl stress conditions, *P*. *occultum* colonization distinctly up-regulated *SlPIP1;5*, *SlPIP2;4*, *SlPIP2;6*, *SlPIP2;10*, and *SlPIP2;11* expressions by 0.47-, 0.63-, 0.48-, and 0.87-fold, respectively, along with no change in *SlPIP1;2*, *SlPIP1;3*, *SlPIP2;1*, *SlPIP2;8*, *SlPIP2;9*, and *SlPIP2;12*, and down-regulation of *SlPIP1;2*, compared with non-fungus colonization. Such results suggested that AMF diversifies to regulate the expression of host *PIPs* in response to NaCl stress, which is consistent with the results of Chen et al. [34] in salt-stressed black locust *PIPs* in response to AMF inoculation. The maintenance of the higher expression of *PIPs* in mycorrhizal plants versus non-mycorrhizal plants under NaCl stress would mean that more water could be taken up from saline soil conditions in mycorrhizal plants [35], thus enhancing the salt tolerance of host plants. 

### 3.5. Effects of AMF on TIPs’ Expressions in Leaves of Tomato under NaCl Stress

**Figure 4:**
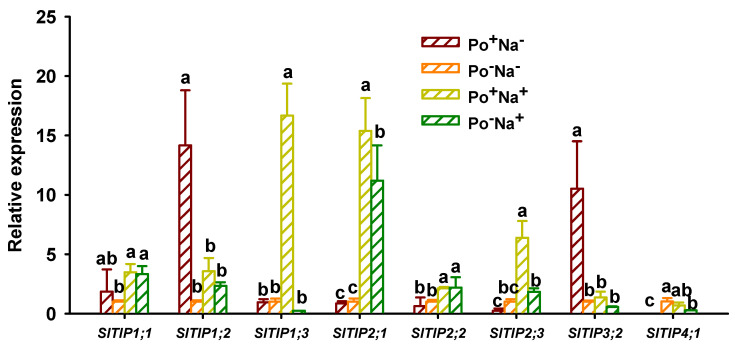
Changes in leaf *SlTIP1* and *SlTIP2* expressions of tomato plants colonized by *Paraglomus occultum* under 0 and 150 mM NaCl stress. Data (means ± SD, *n* = 3) followed by different letters on the bar indicate significant differences among treatments at the 0.05 level. See Table 1 for abbreviations.

Tonoplast intrinsic proteins (*TIPs*) in plants are localized in the tonoplast of cells and can transport a variety of small solutes, including water, hydrogen peroxide, ammonia, etc.; thus, they are associated with salt stress tolerance [17,33,36]. In this study, NaCl stress triggered *SlTIP1;1*, *SlTIP2;1*, and *SlTIP2;2* expressions in non-mycorrhizal plants, coupled with the down-regulation of *SlTIP4;1* and no change in *SlTIP1;2*, *SlTIP1;3*, *SlTIP2;3*, and *SlTIP3;2* expressions (Figure 4). Similarly, in mycorrhizal plants, NaCl stress distinctly induced the up-regulated expressions of *SlTIP1;3*, *SlTIP2;1*, *SlTIP2;2*, *SlTIP2;3*, and *SlTIP4;1*, followed by no change in *SlTIP1;1*, and the down-regulated expressions of *SlTIP1;2* and *SlTIP3;2*. A greater number of up-regulated *TIPs* in inoculated versus uninoculated plants under NaCl stress means that AMF-inoculated plants can tolerate NaCl stress by the up-regulated expression of more *TIPs*. Under non-NaCl stress conditions, *P*. *occultum* colonization significantly increased *SlTIP1;2* and *SlTIP3;2* expressions by 13.10- and 9.48-fold, suggesting that mycorrhizae under non-saline conditions mainly regulate the expression patterns of *SlTIP1;2* and *SlTIP3;2*. Moreover, *TIP1;2* was confirmed to transport hydrogen peroxide [37], suggesting that mycorrhizal effects on tomato plants under non-NaCl stress conditions may be associated with the balance of reactive oxygen species. In trifoliate orange, *P*. *occultum* colonization dramatically up-regulated *PtTIP4;1* and *PtTIP5;1* expressions under non-NaCl stress conditions and *PtTIP4;1* expressions under NaCl stress conditions [38]. This suggests that mycorrhiza-modulated *TIPs* expressions depend on the host plant species.

### 3.6. Effects of AMF on SOS Expressions in Leaves of Tomato under NaCl Stress

**Figure 5:**
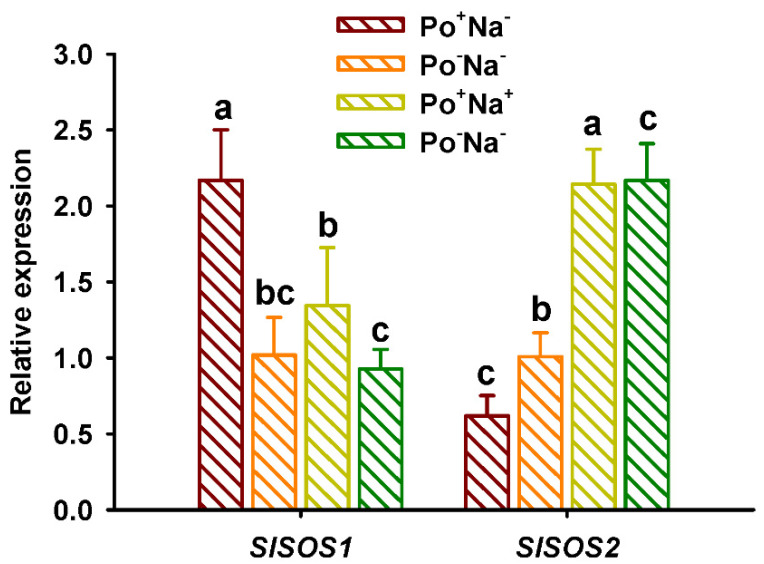
Changes in leaf *SlSOS1* and *SlSOS2* expressions of tomato plants colonized by *Paraglomus occultum* under 0 and 150 mM NaCl stress. Data (means ± SD, *n* = 3) followed by different letters on the bar indicate significant differences among treatments at the 0.05 level. See Table 1 for abbreviations.

NaCl stress up-regulated *SlSOS2* expressions of mycorrhizal and non-mycorrhizal plants, while it inhibited *SlSOS1* expressions of mycorrhizal plants (Figure 5). Belver et al. [39] observed that *SlSOS2* over-expression could raise the exchange of Na^+^/H^+^ at the plasma membrane load Na^+^ into the xylem and the compartmentalization of Na^+^ and K^+^. This suggests that the tomato variety tolerates salt stress mainly by activating *SlSOS2*, but not *SlSOS1*. On the other hand, *P*. *occultum* significantly up-regulated *SlSOS1* expressions by 1.13- and 0.45-fold under non-NaCl and NaCl conditions, respectively. However, for *SlSOS2*, *P*. *occultum* down-regulated its expressions by 0.39-fold under non-NaCl conditions but did not alter its expressions under NaCl conditions. Porcel et al. [40] also reported the up-regulation of *OsSOS1* expressions in rice subjected to salt stress. Under 100 mM NaCl conditions, *Funneliformis mosseae* inoculation up-regulated *SlSOS1* expressions in shoots of *Suaeda salsa* [41]. It is known that *SOS1* is the Na^+^/H^+^ exchanger in the plasma membrane that controls the extrusion and distribution of Na^+^ in tomatoes exposed to salt stress [42]. It was concluded that AMF activated expressions of *SlSOS1* to tolerate salt stress in tomato plants.

## 4. Conclusions

In this study, *P*. *occultum* inoculation was able to improve plant growth as well as nutritional status (e.g., Nbi), and also showed the higher expression of *SlSOS1,* as well as the diverse regulation of *SlPIPs’* expression and higher number of up-regulated *SlTIPs* on inoculated versus uninoculated tomato plants under NaCl stress. This implies that *P*. *occultum*-inoculated tomato plants had higher salt tolerance than uninoculated plants; therefore, *P*. *occultum* has good potential for application in tomatoes under greenhouse and salt stress. However, how *SlSOS1* is activated by *P*. *occultum* and how its signal is transmitted under mycorrhization conditions have yet to be further investigated.

## Figures and Tables

**Table 1:** Changes in root colonization and plant growth parameters of tomato plants colonized by *Paraglomus occultum* under 0 and 150 mM NaCl stress.

Treatments	Root Mycorrhizal Colonization (%)	Plant Height (cm)	Stem Diameter (cm)	Total Plant Biomass (g/plant)
Po^+^Na^−^	49.1 ± 7.6 a	50.6 ± 5.2 a	2.4 ± 0.2 a	18.52 ± 1.49 a
Po^−^Na^−^	0 c	47.0 ± 4.1 a	2.0 ± 0.3 b	12.51 ± 1.39 b
Po^+^Na^+^	38.6 ± 7.1 b	38.5 ± 4.0 b	2.6 ± 0.3 a	12.47 ± 1.13 b
Po^−^Na^+^	0 c	27.4 ± 2.7 c	1.9 ± 0.1 b	8.70 ± 0.71 c

Note: Data (means ± SD, *n* = 6) followed by different letters in the column indicate significant differences among treatments at the 0.05 level. Abbreviations: Po^+^Na^−^, tomato plants inoculated with *Paraglomus occultum* under 0 mM NaCl conditions; Po^−^Na^−^, tomato plants inoculated without *P*. *occultum* under 0 mM NaCl conditions; Po^+^Na^+^, tomato plants inoculated with *P*. *occultum* under 150 mM NaCl conditions; Po^−^Na^+^, tomato plants inoculated without *P*. *occultum* under 150 mM NaCl conditions.

## Data Availability

All the data supporting the findings of this study are included in this article.

## Supplementary Materials

The following supporting information can be downloaded at: https://www.mdpi.com/article/10.3390/jof8121305/s1, Table S1: Specific primer sequences of genes used for qRT-PCR.
