# Supplementary material for: Mycorrhizal Effects on Growth and Expressions of Stress-Responsive Genes (aquaporins and SOSs) of Tomato under Salt Stress
Source: J Fungi (Basel). 2022 Dec 16;8(12):1305. doi: 10.3390/jof8121305 (PMC9786897; doi:10.3390/jof8121305)
Supplement: Supplementary file 1 [file jof-08-01305-s001.zip › jof-2056232-supplementary.pdf]

Supplementary Material Table S1 Specific primer sequences of genes used for qRT-PCR.

Gene names	Accession number	Primer sequences (5'→3')
β-Actin	GQ339765	F: GTCCTCTTCCAGCCATCCA
		R: ACCACTGAGCACAATGTTACCG
SlPIP1;1	Solyc08g008050	F: ACCATCAAATAATCATCAGAGCA
		R: AGGATAAAATAAAAATTATTTTCAT
SlPIP1;2	Solyc01g094690	F: TAGAGACTCCCATGTCCCTATTC
		R: CTAGGCTTCTAGCAGGGTTAATG
SlPIP1;3	Solyc12g056220	F: GGTGTTGTGAAGGGTTTTATGGTT
		R: ACCCAGAAAATCCAGTGGTCATCC
SlPIP1;5	Solyc08g081190	F: CTATCATCTACAACGACGAGCA
		R: CATTGAAGGAGAAACTTGAACA
SlPIP2;1	Solyc09g007770	F: CACATTAACCCTGCTGTTACATTC
		R: CAACCACAAATGGCTCCTAAAC
SlPIP2;4	Solyc06g011350	F: ACGTACCCGTGTTGGCACCTCTTCC
		R: ATGTTCGTCCCACGCCTTGTCACC
SlPIP2;6	Solyc11g069430	F: TACTCCGCAAAGGATTACACTGAT
		R: AGCCCAAGCAATACCAAGTAAACC
SlPIP2;8	Solyc01g111660	F: ATTCCCATATCCCTGTGTTGGCTCC
		R: AGCTGCAGCTCTCAAAATGTATTGG
SlPIP2;9	Solyc10g055630	F: TCTTCTCTGCTACTGACCCTAA
		R: GTGGCCAAATGAACCATGAAA
SlPIP2;10	Solyc09g007760	F: CACATTAACCCTGCTGTTACATTC
		R: CAACCACAAATGGCTCCTAAAC
SlPIP2;11	Solyc02g083510	F: GTCCTCTTCCAGCCATCCA
		R: ACCACTGAGCACAATGTTACCG
SlPIP2;12	Solyc05g055990	F: ATACCCAACGTGTAGCATCACTCTC
		R: CCAGCAGTGGAATACACGAGAACA
SlTIP1;1	Solyc06g074820	F: TCATCACTCCCCAACTTGTGCC
		R: AAAGCCATACCAGAACCCTGACCT
SlTIP1;2	Solyc06g075650	F: ATCCATAGCACATGCCTTTGCCCTT
		R: CCGATGTTTCCAGTCCACCAGTAG
SlTIP1;3	Solyc10g083880	F: CTATTCGTAGCGGTTTCGGTTG
		R: TTGTTCCCAAACTACCCTTCTT
SlTIP2;1	Solyc12g044330	F: TGACTGGAGGAATGGCGGTT
		R: ACCACAGCGGGTCCAAATGA
SlTIP2;2	Solyc03g120470	F: GATTCATTCAGCGTTGTCTCTCTT
		R: AAACGGCTACGAATAGAGCAAATC
SlTIP2;3	Solyc06g060760	F: AATGGTGAAGATTGCCTTTGGTAG
		R: TCAAATGTCCACCTGAGATGTTAG
SlTIP3;2	Solyc03g019820	F: GCTGATTTATTGGTGTTATGGCTATG
		R: AGCAAGAACAGAGCCTTCACCG
SlTIP4;1	Solyc08g066840	F: TTATTCGTAAAATCAGTTTCATCA
		R: CAAGCAGCAACAGAAGCAAGTAAT

SlSOS1	AJ717346	F: GTGCAGTACAGATGCTTTTACTTG<br>R: AGGGCCACAACAGCCACA
SlSOS2	AJ717348	F: ATTTCCCGCCAACCTGCTAA<br>R: TGCCGTTACCCCCTCAATTC
